# Emergence of cutaneous Rosai–Dorfman disease during immunosuppressive treatment of follicular B-cell lymphoma: A case report

**DOI:** 10.1177/2050313X211046455

**Published:** 2021-09-16

**Authors:** Sharon L Kipfer, Michael Samycia, Carolyn J Shiau

**Affiliations:** 1 The University of British Columbia, Vancouver, BC, Canada; 2 Department of Dermatology and Skin Science, The University of British Columbia, Vancouver, BC, Canada; 3 Department of Pathology, Royal Columbian Hospital, New Westminster, BC, Canada; 4 Department of Pathology and Laboratory Medicine, The University of British Columbia, Vancouver, BC, Canada

**Keywords:** Case report, cutaneous Rosai–Dorfman disease, lymphoma, myelodysplastic syndrome, chemotherapy

## Abstract

**Background:**

Sinus histiocytosis with massive lymphadenopathy, also known as Rosai–Dorfman disease, is a rare proliferation of non-Langerhans histiocytes. Cutaneous Rosai–Dorfman disease is a rare subtype of Rosai–Dorfman disease limited to the skin with variable clinical presentation.

**Case summary:**

A 59-year-old female with a history of osteoarthritis, hypothyroidism, and follicular B-cell lymphoma presented with pruritic, erythematous, dome-shaped papules that developed while on chemotherapy treatment. During cutaneous disease progression, the patient was further diagnosed with myelodysplastic syndrome. Histology review revealed patchy staining for S100 in the CD68+ histiocytes within the dermis with no enlarged histiocytes or emperipolesis. Given the absence of other findings, this was interpreted as cutaneous Rosai–Dorfman disease.

**Conclusion:**

There is still little known about the aetiology and pathogenesis of cutaneous Rosai–Dorfman disease. Non-specific immunohistochemistry in the midst of lymphoma, immunosuppressive treatments, and myelodysplastic syndrome produced a blurred diagnostic picture and delayed appropriate treatment, highlighting the diagnostic challenges of cutaneous Rosai–Dorfman disease.

## Introduction

Sinus histiocytosis with massive lymphadenopathy, also known as Rosai–Dorfman disease (RDD), is a rare proliferation of non-Langerhans histiocytes, most commonly involving lymph node sinuses. It was first described as a unique entity in 1969 by Rosai and Dorfman, presenting insidiously with prominent painless cervical lymphadenopathy, leukocytosis, elevated erythrocyte sedimentation rate, fever, anaemia, and hypergammaglobulinaemia.^1^ A rare subtype of RDD, known as cutaneous Rosai–Dorfman disease (CRDD), involves histiocytic proliferation limited to the skin, without concomitant involvement of other sites. The clinical presentation of CRDD varies greatly and as such is often difficult to diagnose.^2,3^ The majority of patients present with multiple lesions, commonly nodules, plaques, and/or papules, being protean in nature. The aetiology and pathogenesis of the systemic and purely cutaneous forms of RDD remain unknown, although there have been numerous proposed theories, including monocyte stimulation by macrophage colony stimulating factor leading to histiocytes in CRDD, and infectious trigger such as Epstein–Barr virus (EBV), HIV, herpes simplex virus (HSV), and human herpesvirus-6 in the presence of immune system anomalies such as uveitis, Crohn’s disease, Sjogren’s syndrome, factor XII deficiency, and lupus erythematosus.^4^ Despite the uncertain origin of presentation, immunohistochemistry allows for recognition and diagnosis of this rare disease.^2,4^ Classic histological findings of CRDD include large foamy histiocytes, emperipolesis, and immunophenotype S100+, CD68+, and CD1a−.^4^

CRDD is typically self-limiting and resolves without need for treatment.^2,4,8^ In a study evaluating lesion evolution, papules and plaques were often seen to form nodules with satellite lesions, which developed into fibrotic plaques before spontaneous resolution.^8^ CRDD generally remains confined to the skin; however, it does frequently recur.^6^ In patients with persistent lesions, symptomatic lesions, or systemic progression, treatment is indicated.^5,7^ Surgical excision of a localized lesion is the most effective and curative treatment. Non-surgical treatments include intralesional or oral steroids, chemotherapy, radiation, cryotherapy, and tyrosine kinase inhibitors,^4,5,7^ although the efficacy of these treatment options is still being understood.^2^

## Case report

**Figure 1.**
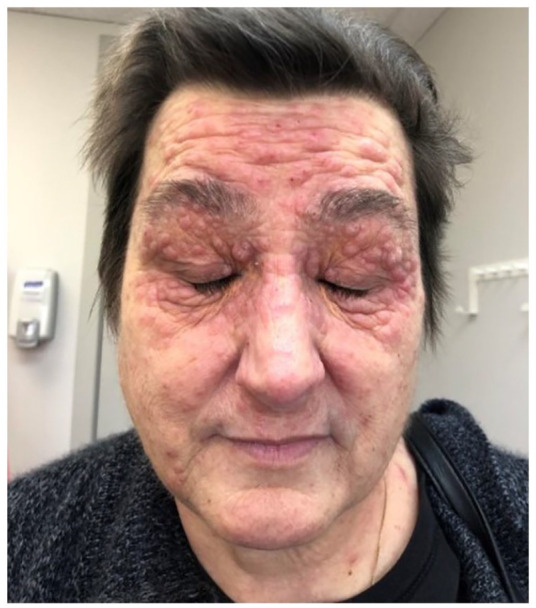
Patient with facial lesions consistent with her typical presentation.

A 59-year-old female with a history of osteoarthritis, hypothyroidism, and follicular B-cell lymphoma presents with a rash on her face and body. This rash had initially developed on treatment with bendamustine and rituximab chemotherapy for her recent lymphoma diagnosis. Pruritic, erythematous, dome-shaped papules with crusting were present diffusely on the patient’s back, neck, and face, particularly forehead, perinasal, and periorbital areas (Figure 1). Bendamustine was discontinued due to cytopenia and worsening symptoms, and treatment was switched to R-CHOP (rituximab, cyclophophamide, doxorubicin hydrochloride, vincristine sulfate, prednisone). Change in treatment seemed to improve the rash on her body with no change in facial rash.

**Figure 2.**
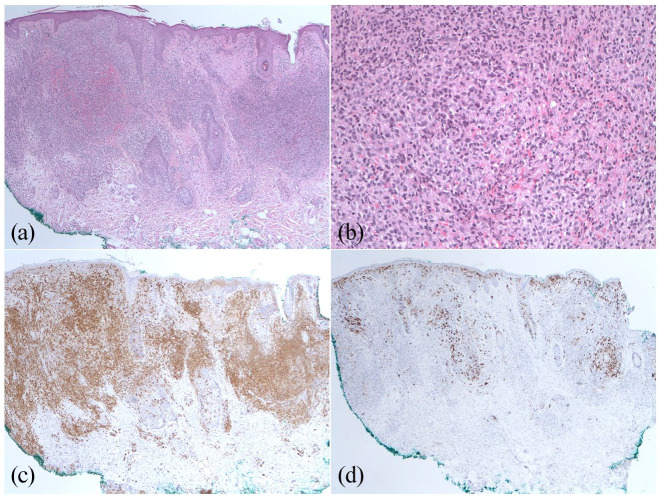
(a) Histological finding of initial biopsy showing dense lymphohistiocytic inflammation around the hair follicle. (b) Admixed small histiocytes and lymphocytes were noted with no enlarged cells and no emperipolesis. Immunohistochemistry for dermal-based histiocytes showed (c) increased cytoplasmic staining for S100 and (d) no staining for CD1a. The background epidermal Langerhans cells are positive as internal control for both S100 and CD1a.

Initial skin biopsy demonstrated dermal lymphohistiocytic infiltrate with a proposed differential diagnosis including granulomatous rosacea or granulomatous folliculitis (Figure 2(a) and (b)). A trial of minocycline was initiated but discontinued due to side effects. Treatment was switched to doxycycline 100 mg daily in conjunction with metronidazole 1% cream with minimal improvement.

Repeat biopsy of a lesion on the forehead revealed dense superficial perifollicular lymphohistiocytic infiltrate in the superficial and mid dermis, suggestive of granulomatous rosacea. Treatment was again switched to erythromycin 500 mg PO twice a day (BID) and clobetasol 0.05% ointment BID to the face as the patient’s primary symptom was extreme pruritus. This led to significant improvement. Clobetasol was eventually switched to Protopic 0.1% ointment.

Given the persistent facial lesions and complex clinical picture, the case was reviewed. There was no involvement of the skin by follicular lymphoma on morphology. Immunohistochemistry showed patchy staining for S100 in the histiocytes within the dermis with no staining for CD1a (Figure 2(c) and (d)). Given the unusual immunoprofile, cutaneous involvement by a non-Langerhans cell histiocytosis was proposed, and a diagnosis of CRDD was made. During this cutaneous disease progression, the patient was further diagnosed with myelodysplastic syndrome (MDS).

This patient’s CRDD remains as scattered, minimally elevated papules on the forehead with some excoriated papules on the face and xanthelasma periocularly. She has since been managed with erythromycin (oral and topical), loratadine, hydroxyzine, tacrolimus ointment, clobetasol scalp lotion, and Stieprox shampoo.

## Discussion

CRDD presents as cutaneous lesions of papules and/or nodules that are frequently discoloured with no predilection to a specific site and with no systemic involvement (Brenn, Calonje and Granter, 2002). There have been many proposed aetiologies and pathogeneses, including infectious disease, immunomodulation, and neoplastic origins, but no clear path has been determined (Brenn, Calonje and Granter, 2002), (Gameiro, Gouveia and Cardoso, 2016), (Sewon, Amagai and Bruckner, 2019), (Wang, Chen and Liu, 2005). Given the high degree of variability in clinical findings and histopathology, the differential diagnosis for CRDD remains broad and may be clinically indistinguishable from other tumorous, inflammatory, or histiocytic presentations (Sewon, Amagai and Bruckner, 2019).

Our patient demonstrated some typical cutaneous findings seen in CRDD: erythematous papules present diffusely on the face with subsequent lesions on the trunk and limbs. However, additional cutaneous findings less commonly seen in CRDD of crusting, pruritic symptoms, and non-resolution blurred the diagnosis of CRDD. Given the non-specific clinical presentation, the complication of disease presentation following chemotherapy treatment, as well as concurrent systemic diseases of follicular B-cell lymphoma and MDS, the clinical picture was rendered non-specific and a provisional diagnosis of granulomatous rosacea was made. The typical histological features of CRDD (enlarged histiocytes with emperipolesis) were not present in this case. However, in the absence of any other findings, the presence of histiocytes with S100+ and CD1a− immunoprofile was interpreted to be highly suspicious for CRDD.

There have been multiple reports of RDD, both systemic and purely cutaneous forms, occurring in patients concurrently with Hodgkin’s, B-cell, and T-cell lymphoma.^2,4,6^ The documented comorbidity of lymphoma and RDD has led to speculation on a common pathogenic process leading to evolution of these diseases concurrently.^4,6^ Iatrogenic immune dysregulation is suggested to lead to hyperplasia within lymph nodes, leading to the lymphoma.^4^ Others speculate a similar pathogenic process occurring with these two distinct entities, resulting in a variant phenotypic expression, being either RDD or lymphoma, with occasional concurrent expression.^6^

The unclear clinical picture, atypical histological findings, and comorbid state of this patient highlight the diagnostic challenges associated with CRDD. Given the rarity of CRDD, the lack of knowledge on pathogenesis, the variability in presentation, and the variation in treatment options/efficacy, more work is required on this disease in order to diagnose and manage these patients effectively.
